# Detection and genetic characterization of feline bocavirus in Northeast China

**DOI:** 10.1186/s12985-018-1034-3

**Published:** 2018-08-08

**Authors:** Shushuai Yi, Jiangting Niu, Hualei Wang, Guoying Dong, Yanli Zhao, Hao Dong, Yanbing Guo, Kai Wang, Guixue Hu

**Affiliations:** 1 College of Animal Science and Technology, Jilin Agricultural University, Changchun, 130118 Jilin Province China; 2 Key Laboratory of Jilin Province for Zoonosis Prevention and Control, Military Veterinary Research Institute, Academy of Military Medical Sciences, Changchun, 130122 China; 3 College of Global Change and Earth System Science, Beijing Normal University, Haidian, Beijing, 100875 China; 4 Library, Jilin Agricultural University, Changchun, 130118 Jilin Province China; 5 College of Life Science and Technology, Jilin Agricultural University, Changchun, 130118 Jilin Province China; 6 Jilin Institute of Animal Husbandry and Veterinary Science, Changchun, 130062 Jilin Province China

**Keywords:** Feline bocavirus, Genotype, Genetic characterization, Complete genome

## Abstract

**Background:**

Bocaviruses have been reported to cause respiratory tract infection and gastroenteritis in most animal species. In cats, different genotype bocaviruses have been identified in USA, Japan, Hong Kong and Portugal. However, the clear relationship between the clinical symptoms and FBoV infection is unknown, and the prevalence of FBoV and the distribution of FBoV genotypes in China are still unclear.

**Results:**

In this study, 197 fecal samples from cats with diarrhea (*n* = 105) and normal cats (*n* = 92) were collected in different regions between January 2016 and November 2017 and investigated using PCR targeting different FBoV genotypes. Screening results showed that 51 of 197 samples (25.9%) were positive for FBoV, and a higher positive rate was observed in cats with diarrhea (33.3%, 35/105) than in normal cats (17.4%, 16/92). Of these FBoV-positive samples, 35 were identified as FBoV-1, 12 as FBoV-2 and 4 as coinfection of FBoV-1 and FBoV-2. A phylogenetic analysis based on partial NS1 gene indicated that 24 sequences from randomly selected FBoV-positive samples were divided into 2 different FBoV groups: FBoV-1 and FBoV-2. Furthermore, 6 strains were randomly selected, and the complete genome was sequenced and analyzed. These strains exhibited the typical genome organization of bocavirus and were closely related to FBoV. Two FBoV-2 identified strains shared high homologies with FBoV-2 reference strains based on the complete genome and entire encoding gene, but lower identities were exhibited in the NP1 and VP1 regions for the other 4 FBoV-1 identified strains compared with FBoV-1 reference strains.

**Conclusion:**

These findings demonstrate that genetically diverse FBoV-1 and FBoV-2 widely circulate in cats in Northeast China and that FBoV-1 is more prevalent. The high prevalence of FBoV in cats with diarrhea symptoms suggests that FBoV infection may be associated with diarrhea in cats.

**Electronic supplementary material:**

The online version of this article (10.1186/s12985-018-1034-3) contains supplementary material, which is available to authorized users.

## Background

Bocavirus, a member of the genus *Bocavirus* which belongs to the subfamily *Parvovirinae* in the family *Parvoviridae*, is a non-enveloped and isometric icosahedral virus with a diameter of approximately 25–26 nm [1, 2]. The bocavirus genome, which consists of an approximately 5.5 kb linear single-stranded DNA, possesses three open reading frames (ORFs): ORF1, ORF2 and ORF3, encoding non-structural protein NS1, viral capsid proteins VP1/VP2 and nuclear phosphoprotein NP1, respectively [1, 3]. For bocavirus, the NP1 protein is critical in viral replication and viral survival, although the data are not very strong, and is unique for bocavirus, distinguishing it from other members in the family *Parvoviridae* [4, 5]. According to the latest ICTV report [2, 6], the genus *Bocavirus* is divided into twelve species: *Carnivore bocaparvovirus* 1–3, *Primate bocaparvovirus* 1–2, *Ungulate bocaparvovirus* 1–5 and *Pinniped bocaparvovirus* 1–2, which contain canine minute virus (MVC), canine bocavirus (CBoV), feline bocavirus (FBoV), human bocavirus (HBoV), gorilla bocavirus (GBoV), bovine parvovirus (BPV), porcine bocavirus (PBoV) and California sea lion bocavirus (CslBoV) [7–11].

Abundant previous research has reported that different clinical symptoms are caused by bocaviruses in homologous hosts. For example, acute lower respiratory tract infections caused by HBoV have been reported in many countries [12]. HBoV has also been found to cause gastroenteritis in children in recent reports [13]. Respiratory tract symptoms and diarrhea have been reported in weanling piglets infected with PBoV [8, 14]. Furthermore, it has been confirmed that CBoV is related to gastroenteritis in dogs [15]. Recent reports showed that the genome of CBoV has been detected in the respiratory tract, feces, lymphonodus, liver and blood of dogs, suggesting that CBoV could cause systemic infections in dogs [16, 17].

Feline bocavirus (FBoV) belongs to the species *Carnivore bocaparvovirus* 3 in the genus *Bocavirus*. In 2012, a novel bocavirus designated FBoV was firstly detected in fecal, kidney, nasal and blood samples of cats with or without clinical symptoms in Hong Kong [9]. Since then, FBoV-2 (POR1) and FBoV-3 (FBD1) were identified in fecal samples of cats using high-throughput sequencing technology [18, 19]. At present, FBoVs have been classified into three genotypes, FBoV-1, − 2 and − 3, and have been reported in the USA, Japan and Portugal [9, 18–20]. Recently, Liu et al. first detected FBoV-1 (HRB2015-LDF) in fecal samples from cats with severe enteritis in mainland China [21]. However, the clear relationship between the gastrointestinal symptoms and the prevalence of FBoV is unknown, and the prevalence of FBoV and the distribution of FBoV genotypes in China are still unclear. In this study, we investigated the prevalence of different FBoV genotypes in Northeast China, and determined the relationship between the presence of FBoV and diarrhea in cats. We also examined the genetic characteristics of FBoVs identified in the present study and discussed the evolutionary relationships between FBoV and other bocaviruses based on the NS1 gene and complete bocavirus genome.

## Methods

### Sample collection and DNA extraction

Fecal samples were collected from private veterinary clinics and animal shelter centers in Shenyang, Jinzhou, Changchun, Jilin and Harbin in Northeast China between January 2016 and November 2017. Fresh feces were immediately collected using aseptic swab after defecation, and placed in RNase-free tubes. Samples collection was reviewed and approved by the Animal Care and Use Committee of Jilin Agricultural University, and was consented by client-owner. In total, 197 fecal samples were collected from 105 cats with diarrhea (the main clinical feature is acting depressed, anorexia, and the excretion of soft feces, watery or bloody stools.) and 92 healthy cats without any clinical symptoms and were examined in the present study. Approximately 1 g of fecal sample was homogenized in 5 ml of phosphate-buffered saline solution (PBS, pH = 7.4) using a vortex oscillator and then centrifuged at 10,000×g for 10 min at 4 °C to collect the supernatant. Each sample’s genome DNA was extracted from 250 μl of supernatant using the Viral DNA Extraction Kit I (OMEGA, China) according to the manufacturer’s instructions and then stored at − 70 °C until further use.

### Detection of FBoVs in clinical samples

The genomic DNA extracted from fecal samples was analyzed for FBoVs using polymerase chain reaction (PCR) assays with three different primer pairs for FBoV-1, − 2 and − 3 as described in previous papers [9, 18, 19]. PCR was performed in 25 μl of reaction mixture containing 2 μl of template DNA, 12.5 μl of Premix *Ex* Taq (TAKARA, Japan), 1 μl of 10 pM forward and reverse primer and 8.5 μl of RNase free water using a thermocycler (BIOER, China). The primer sequences are shown in Table 1, and the amplification conditions were described previously. The sizes of the amplified DNA fragments were 133 bp for FBoV-1, 331 bp for FBoV-2 and 546 bp for FBoV-3. The amplified products were separated after electrophoresis on 1.5% agarose gels at 160 V for 20 min and visualized using a gel documentation system (Wealtec, USA). The prevalence of FBoVs and the distribution of the three genotypes was calculated, and the relationship between the prevalence of FBoVs and diarrhea symptoms in cats was analyzed with a univariable logistic regression model using the statistical program PASW Statistics 19.0. Probability (*p*) values < 0.05 were considered statistically significant.

**Table 1:** Nucleotide sequences of primers used for screening and sequencing in this study

Primer name	Nucleotide sequence (5′-3′)	Product (bp)	Application
FBoV1F	TCTACAAGTGGGACATTGGA	133	Screening for FBoV-1 [11]
FBoV1R	GAGCTTGATTGCATTCACGA		
FBoV2F	TCGTTCGTCTTGGAACATAGC	331	Screening for FBoV-2 [17]
FBoV2R	CAGAGCGTGGATCTGTCTGA		
FBD1L1	TGACTCGTCTGTGGCGGGCT	546	Screening for FBoV-3 [27]
FBD1R1	TCGTTCGTGAGACGCTGCCA		
FBOV1-NS1F	TTTGGGGCTGAAGTCTGCTATGC	705	Sequencing for partial NS1 gene of FBoV-1
FBOV1-NS1R	ATGCGGCTGAGATGTACCTTGACC		
FBOV2-NS1F	TTCGCGGATCCAGCATACACCTAC	963	Sequencing for partial NS1 gene of FBoV-2
FBOV2-NS1R	CTCACAACGGCGCAAGCAGTCTA		
FBoV1-55F^a^	CCGGCGCGATGACGTGTCAG	523	Sequencing for the complete genome of FBoV-1
FBoV1-577R	GCGCCGCATAGCAGACTTCAG		
FBoV1-550F	TTTGGGGCTGAAGTCTGCTATGCG	1695	
FBoV1-2245R	CGATGTCGCCTCGATCGTGYAGTTC		
FBoV1-2177F	ATGAGACCGGAACGTGTATGAGCT	1407	
FBoV1-3583R	GGCGCCCCCTCTTGTCCAT		
FBoV1-3553F	AGACGGCTGGGGATGGACAAGA	1411	
FBoV1-4963R	GGYAGTGTGCCATCAGCCGAATC		
FBoV1-4734F	ATGGCTAGATGGGGTTCAGTG	635	
FBoV1-5368R	GTAGCAGTGTGGAGGGTGTTGTAT		
FBoV2-66F^b^	GATGACGTGTCAGTGTGGGTGTTG	1278	Sequencing for the complete genome of FBoV-2
FBoV2-1343R	ACGTTAGCCACAAATTATCCTCACA		
FBoV2-1306F	CTGCTTGCGCCGTTGTGAGGATAAT	1102	
FBoV2-2407R	GTCTGGAGCACTGCCGCCGTCTG		
FBoV2-2384F	ACAGACGGCGGCAGTGCTCCAGAC	1315	
FBoV2-3698R	CCATGCCCGCCACCCCCAGAG		
FBoV2-3653F	GTGGGGGTGGCAGCGGTGTTGG	1014	
FBoV2-4666R	CGCCTCCCCTGTCTGGTATGTTGG		
FBoV2-4219F	CTAAACGCAGATAATGTGGAAAAA	1176	
FBoV2-5394R	CAGTGTGGGTGATGTTGAGAAG		

^a^The primer positions refer to the full-length genome of FBoV genotype 1 strain FBD2 (GenBank accession no. KM017745); ^b^The primer positions refer to the full-length genome of FBoV genotype 2 strain POR1 (GenBank accession no. KF792837)

### Sequencing of partial NS1 gene

To further determine the genotype of FBoVs detected in this study, 21 FBoV-positive samples were randomly selected for amplification and sequencing of partial NS1 gene. By aligning the nucleotide sequence of the NS1 gene among different FBoV genotype strains, two primer sets in the same region that could identify FBoV genotype and perform phylogenetic analysis, FBoV1-NS1F/FBoV1-NS1R targeted to a 705-bp region of the NS1 gene for FBoV-1 and FBoV2-NS1F/ FBoV2-NS1R to amplify a 963-bp fragment in NS1 gene for FBoV-2 (the primer sequences are shown in Table 1), were designed in the present study. We also optimized the reaction system and amplification conditions of these two primer sets in our study. Thirty nanograms of sample DNA was mixed with a total volume of 45 μl of reaction mixture containing 25 μl of Premix *Ex* Taq (TAKARA, Japan), 2 μl of 10 pM forward and reverse primers and 18 μl of RNase free water, and the amplification was performed with a thermocycler (BIOER, China) using the following conditions: initial denaturation at 94 °C for 5 min, followed by 35 cycles of 94 °C for 1 min, 62 °C for 1 min and 72 °C for 1 min, and a final extension at 72 °C for 7 min. PCR products were visualized on 1.0% agarose gels and purified using an AxyPrep DNA gel extraction kit (Axygen, China) according to the manufacturer’s instructions. The purified DNA was cloned in to a PMD-18 T vector, and positive clones were selected and sent to Sangon Biotech (Shanghai, China) for sequencing. To ensure sequence accuracy, each sample was sequenced using at least three positive clones, and each specific base was sequenced at least four times.

### Amplification of complete genome for FBoVs and sequencing

The full-length genomes of different FBoV genotypes from different regions were also determined using 5 pairs of specific primers for FBoV-1 and 5 pairs for FBoV-2, that we designed in this study. The nucleotide sequences of primers are shown in Table 1. The amplification was carried out in the same reaction mixture as described above, and the reaction conditions were as follows: predenaturation at 94 °C for 5 min, followed by 35 cycles of denaturation at 94 °C for 1 min, annealing at 60 °C for 1 min and extension at 72 °C for 90 s, and a final extension at 72 °C for 7 min. The PCR products were purified and cloned into PMD-18 T vectors according to the same procedures that we used in sequencing the partial NS1 gene. Positive clones were sent to Sangon Biotech (Shanghai, China) for sequencing. The nucleotide sequences were assembled using SeqMan program in the Lasergene 7.0 software (DNASTAR, USA), and the nearly complete FBoV genome sequences have been deposited in GenBank under accession numbers MH155946 – MH155951.

### Genomic, phylogenetic and recombination analyses

Partial NS1 gene sequences generated in this study were compared with those of other feline bocaviruses in GenBank using an online BLAST program (https://blast.ncbi.nlm.nih.gov/Blast.cgi). The NS1 gene sequences alignment between FBoV strains identified in this study and representative bocavirus strains in the GenBank database (Additional file 1) were generated using ClustalW, and a phylogenetic tree based on the partial NS1 gene was constructed using the neighbor-joining (NJ) method with 1000 bootstrap replicates in MEGA 7.0 software.

For each complete genome that we obtained from FBoV-positive samples in the present study, the ORFs were predicted using two online programs: ORF finder (https://www.ncbi.nlm.nih.gov/orffinder/) and Gene MarkS (http://opal.biology.gatech.edu/GeneMark/genemarks.cgi). Phylogenetic tree of FBoVs identified in this study with different FBoV genotypes reference strains and other bocaviruses isolated from different species (Additional file 1) based on complete genomic nucleotide sequences was obtained using the neighbor-joining method, and 1000 replications were set for a bootstrap analysis. Maximum-likelihood (ML) phylogenetic analysis was also performed to confirm the results. SimPlot 3.5 software was used to determine the similarities of the FBoV genomes. The average percentage of nucleotide and amino acid identities was calculated using the MegAlign program. For phylogenetic analyses of NS1, NP1 and VP1, we constructed phylogenetic trees based on amino acid sequences. The possible recombination breakpoints were identified using the RDP method in the Recombination Detection Program (RDP) 4.0.

## Results

### Detection of FBoVs in fecal samples

**Fig. 1:**
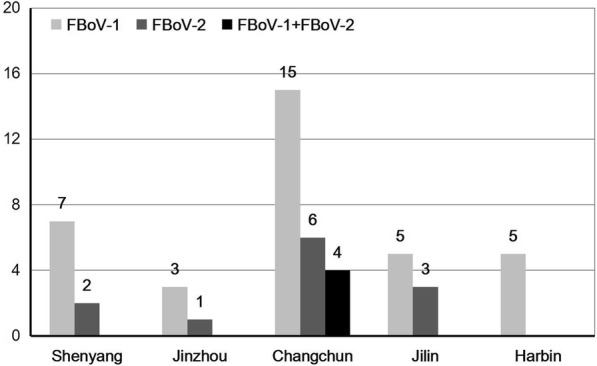
Feline bocavirus positive samples identified in different regions. The number of different genotypes in each region is indicated above the bars

Out of 197 fecal samples, 51 (25.9%) samples were identified to be positive for FBoV using three pairs of specific primers for different FBoV genotypes. Of these FBoV-positive samples, 35 samples were positive for FBoV-1 and 12 were positive for FBoV-2. Interestingly, 4 samples were simultaneously identified as positive for FBoV-1 and FBoV-2 (Table 2). FBoV-1 was most prevalent in Northeast China. Detailed informations for the 51 FBoV-positive samples is shown in Additional file 2. We investigated the relationship between FBoV-infection and diarrhea symptoms, regions, season and residential environment (the sample’s source). The differences in the FBoV prevalence in different regions (19.2–29.4%) and different season (21.6–33.3%) were not significant. Interestingly, the two FBoV genotypes (FBoV-1 and -2) were present in all regions except for Harbin, while all samples that identified as co-infection of FBoV-1 and FBoV-2 were collected from Changchun (Fig. 1). Cats with diarrhea symptoms had a higher FBoV-positive rate (33.3%) than normal cats (17.4%). In the samples collected from animal shelter centers, the prevalence of FBoV was significantly higher than in samples from private veterinary clinics (Table 2). These results suggest that the FBoV infections were related to diarrhea symptoms and residential environments but were not associated with regions.

**Table 2:** Prevalence of feline bocavirus infection in cats in Northeast China

Factor	Category	no. tested	Prevalence (no. positive)	Univariable analysis^a^			The number of different genotype in FBoV- positive samples		
				OR	95% CI	*P* value	FBoV-1	FBoV-2	FBoV-1+ FBoV-2
Region	Shenyang	36	25% (9)	Reference			7/9	2/9	0/9
	Jinzhou	17	23.5% (4)	1.180	0.485–2.874	0.715	3/4	1/4	0/4
	Changchun	85	29.4% (25)	0.714	0.208–2.451	0.593	15/25	6/25	4/25
	Jilin	33	24.2% (8)	1.125	0.384–3.297	0.830	5/8	3/8	0/8
	Harbin	26	19.2% (5)	0.923	0.239–3.564	0.908	5/5	0/5	0/5
Source	PVC	71	16.9% (12)	Reference			10/12	2/12	0/12
	ASC	126	30.9% (39)	2.445	1.160–5.154	0.019	25/39	10/39	4/39
Clinical symptom	Diarrhea	105	33.3% (35)	Reference			26/35	6/35	3/35
	Normal	92	17.4% (16)	2.375	1.210–4.663	0.012	9/16	6/16	1/16
Season	Spring	35	25.7% (9)	Reference			6/9	3/9	0/9
	Summer	51	21.6% (11)	0.679	0.285–1.614	0.380	7/11	3/11	1/11
	Autumn	75	25.3% (19)	0.592	0.248–1.933	0.483	14/19	4/19	1/19
	Winter	36	33.3% (12)	0.550	0.210–1.439	0.223	8/12	2/12	2/12
Total		197	25.9% (51)				35/51	12/51	4/51

*PVC* private veterinary clinic, *ASC* animal shelter center, *OR* odds ratio, *CI* confidence interval, *p value* probability value

^a^The univariable analysis was performed with a univariable logistic regression model using PASW Statistics 19.0. The screening result (positive or negative) was used as the dependent variable, and the factors were used as the covariates

### Partial NS1 gene phylogenetic analysis

**Fig. 2:**
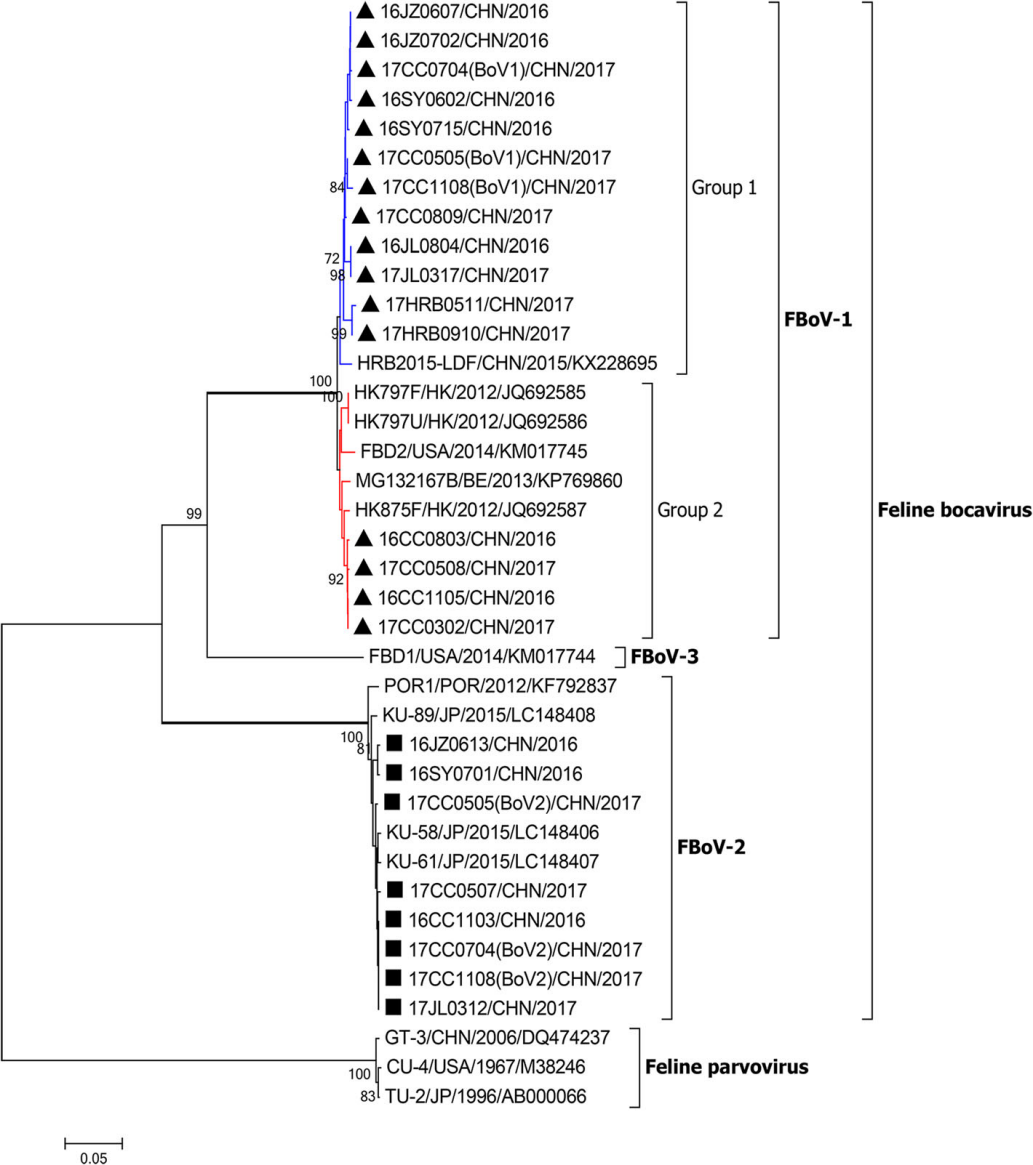
Phylogenetic tree based on partial NS1 gene of feline bocavirus. The phylogenetic relationship of the nucleotide sequences (705 nt) was determined using the neighbor-joining method with 1000 bootstrap replicates using MEGA 7.0 software, and only bootstra*p* values > 70% are displayed above the tree branches. The black triangles indicate strains belonging to FBoV-1 identified in this study, and the black squares indicate strains belonging to FBoV-2 identified in the present study. Group 1 and group2 of FBoV-1 are indicated by blue and red lines, respectively. FBoV, feline bocavirus; BE, Belgium; CHN, China; HK, Hong Kong; POR, Portugal; JP, Japan; USA, United States of America

In this study, 21 FBoV-positive samples, including 13 FBoV-1-positive samples, 5 FBoV-2-positive samples and 3 coinfection samples, collected from different regions were randomly selected for sequencing of partial NS1 genes. Twenty-four nucleotide sequences of partial NS1 genes were obtained and have been been deposited in GenBank under accession numbers MH155928 – MH155951. These sequences were compared with different FBoV genotype reference strains and were compared to each other. Eight strains belonged to the FBoV-2 group and shared a high nucleotide and deduced amino acid identity with each other (98.6–100% nt identity and 99.1–100% aa identity) and with the FBoV-2 reference strain POR1 (98.0 - 99.6% nt identity and 98.3–99.6% aa identity). Sixteen strains belonged to the FBoV-1 group and showed 96.9–100% nt identity and 97.1–100% aa identity to each other, and 96.3–98.6% nt identity and 96.7–98.8% aa identity to the FBoV-1 representative strains HK797U, HRB2015-LDF and FBD2 identified in different countries. In the NJ phylogenetic tree based on the nucleotide sequence of partial NS1 gene, all of the feline bocaviruses could be grouped into three large branches: the FBoV-1, FBoV-2 and FBoV-3 branches. All of the FBoV-1-positive strains were placed in the FBoV-1 branch, and 8 FBoV-2-positive strains were placed in the FBoV-2 branch and displayed a closer relationship to the FBoV-2 strains from Japan (Fig. 2). To confirm our result, we also constructed a ML phylogenetic tree based on partial NS1 gene (Additional file 3). This second analysis also showed coincident grouping with the NJ phylogenetic analysis. Moreover, 16 FBoV-1 strains identified in this study and other FBoV-1 representative strains formed two major groups. Four of these 16 strains (16CC0803, 16CC1105, 17CC0302 and 17CC0508) and other reference strains from the USA, Hong Kong and Belgium were classified in group 2, whereas the other 12 strains clustered together forming group 1 and appeared to be more closely related to strain HRB2015-LDF from Northeast China in 2015 (Fig. 2).

### FBoVs genomic and phylogenetic analysis

**Fig. 3:**
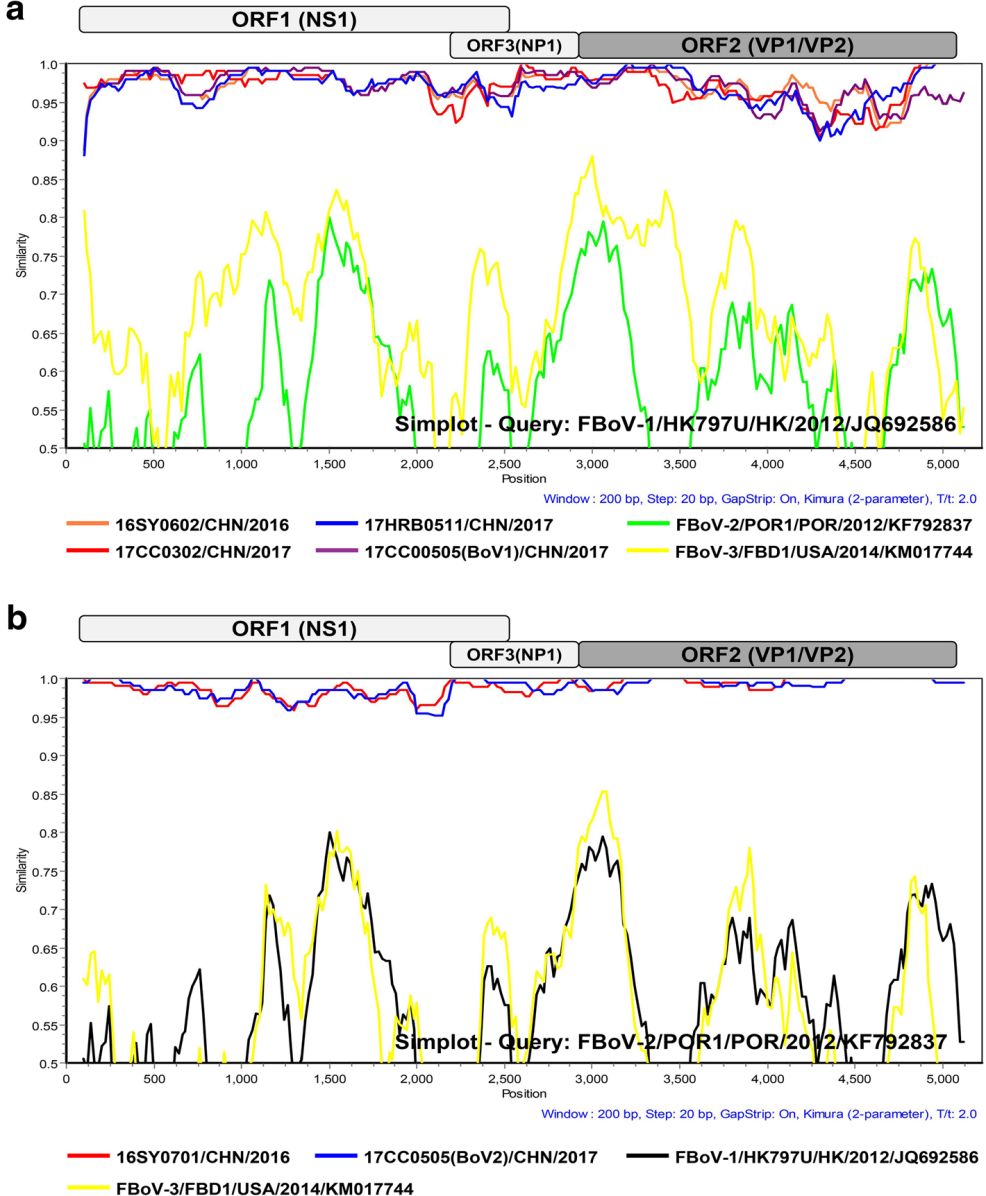
Similarity plot analysis of the complete genomes from different genotype FBoV strains using a Kimura (2-parameter) model with a sliding window of 200 nt and a moving step size of 20 nt. (**a**) The genetic similarity plot of 16SY0602 (orange), 17HRB0511 (blue), 17CC0302 (red), 17CC0505(BoV1) (purple), FBoV-2/POR1 (green), FBoV-3/FBD1 (yellow), and FBoV-1/HK797U as a query sequence. (**b**) The similarity plot of the complete genome of 16SY0701 (red), 17CC0505(BoV2) (blue), FBoV-1/HK797U (black), FBoV-3/FBD1 (yellow), and FBoV-2/POR1 as a query sequence

A genomic analysis was performed using 6 nearly complete genomic sequences obtained from five FBoV-positive samples (included one mixed infection sample) in this study. The analytic results showed that the total genome length was identical for the same FBoV genotype strains: 5314 bp for all strains (16SY0602, 17HRB0511, 17CC0302 and 17CC0505(BoV1)) belonging to the FBoV-1 group and 5329 bp for the two other strains (16SY0701 and 17CC0505(BoV2)) belonging to the FBoV-2 group. Three ORFs (encoding NS1, NP1 and VP1) were predicted in each genome using Gene Marks and ORF Finder. The genetic organization of the full-length genome obtained in this study was coincident with that previously described for FBoV. The complete genome of FBoV-1 strains identified in the present study shared 96.8–98.7% nt identity and 94.9–97.0% aa identity with each other, and shared the closest nt and aa identities of 96.9–97.8% and 94.6–95.9% with two FBoV-1 reference strains (HK797U and HRB2015-LDF), respectively. The amino acid similarities of NS1, NP1 and VP1 genes between the identified FBoV-1 strains and reference strains were 96.9–97.8%, 95.9–97.3% and 94.9–96.9%, respectively. For the two Chinese FBoV-2 strains, the genome similarity was 98.9 and 98.6% at the nt level and aa level, respectively, when compared to each other. Greater identities (98.9–99.0% nt identity and 98.8–98.9% aa identity) were exhibited between the two Chinese FBoV-2 strains and the reference FBoV-2 strain (POR1); furthermore, for the NS1, NP1 and VP1 amino acid levels, the two FBoV-2 strains were 98.8–98.9%, 98.7–99.6% and 99.2–99.6% similar to the reference strain (Additional file 4). To further analyze the characteristics of the full-length genome of the six identified strains, a similarity plot analysis was performed using Simplot. Two prototype strains, HK797U for FBoV-1 and POR1 for FBoV-2, were used as query sequences to analyze the similarities of different genotype strains. 16SY0701 and 17CC0505(BoV2) displayed higher similarities than the other four FBoV-1 strains at the complete genome level when compared to query sequences. The four FBoV-1 strains identified in this study presented greater evolutionary variation in the NP1 and VP1/VP2 regions than in the NS region. However, higher similarities were exhibited in the NP1 and VP1/VP2 regions for the two Chinese FBoV-2 strains compared to each other and the query sequence (Fig. 3). We next investigated the recombination events in FBoV genome of 6 strains identified in this study and 5 different genotype strains deposited in GenBank using the RDP method. The analytical results indicated that no recombination breakpoints were found in any of the Chinese FBoV strains.

**Fig. 4:**
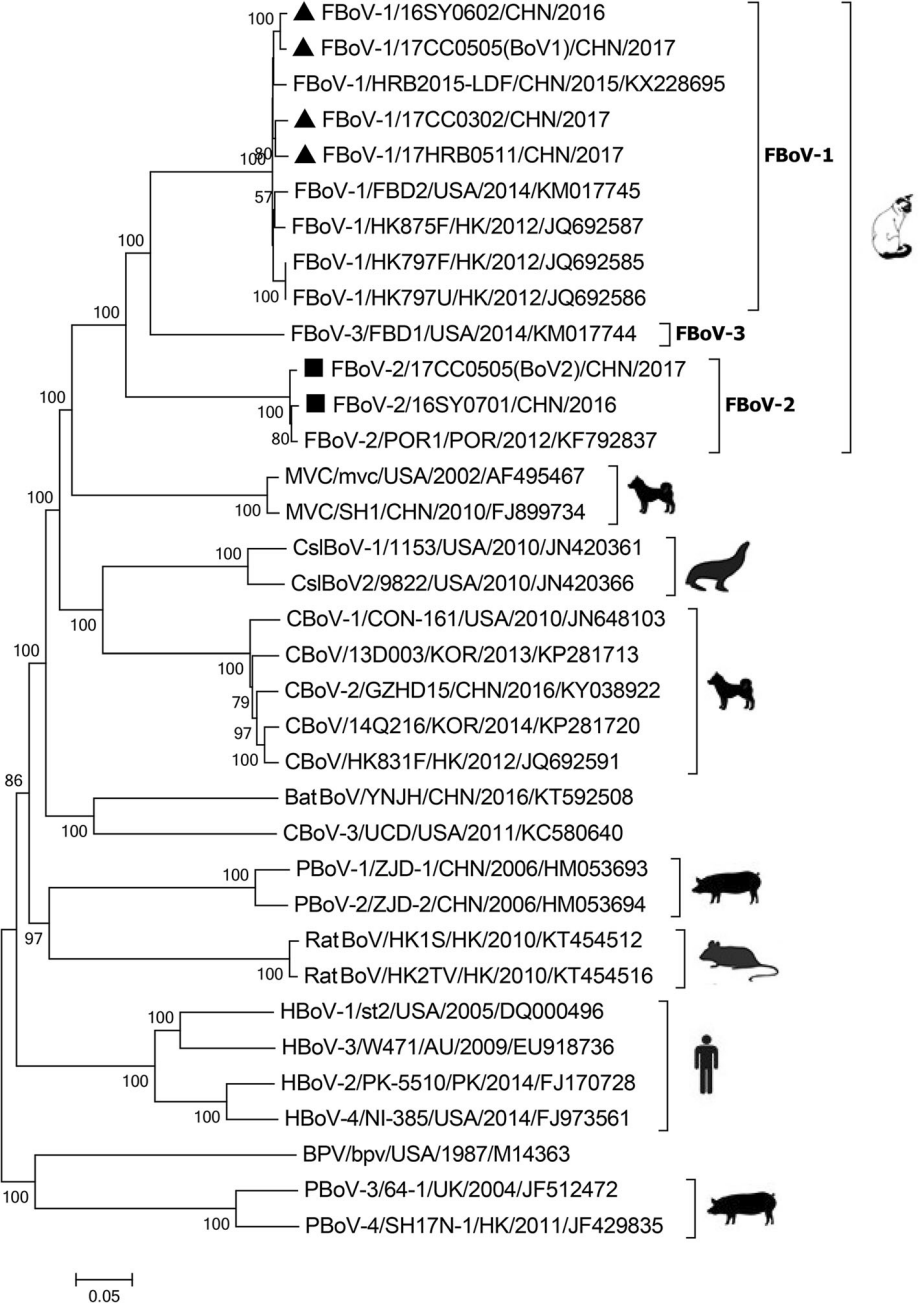
Phylogenetic analysis of bocavirus based on the complete genome. The black triangles and black squares indicate FBoV-1 strains and FBoV-2 strains, respectively, identified in this study. The phylogenetic relationship was determined using the neighbor-joining method with 1000 bootstrap replicates, and only bootstrap values > 50% are displayed above the tree branches. The silhouettes of hosts for different bocaviruses are shown on the branches. BatBoV, bat bocavirus; BPV, bovine parvovirus; CslBoV, California sea lion bocavirus; CBoV, canine bocavirus; FBoV, feline bocavirus; HBoV, human bocavirus; PBoV, porcine bocavirus; RatBoV, rat bocavirus. AU, Australia; CHN, China; HK, HongKong; JP, Japan; KOR, South Korea; POR, Portugal; PK, Pakistan; USA, United States of America; UK, United Kingdom

**Fig. 5:**
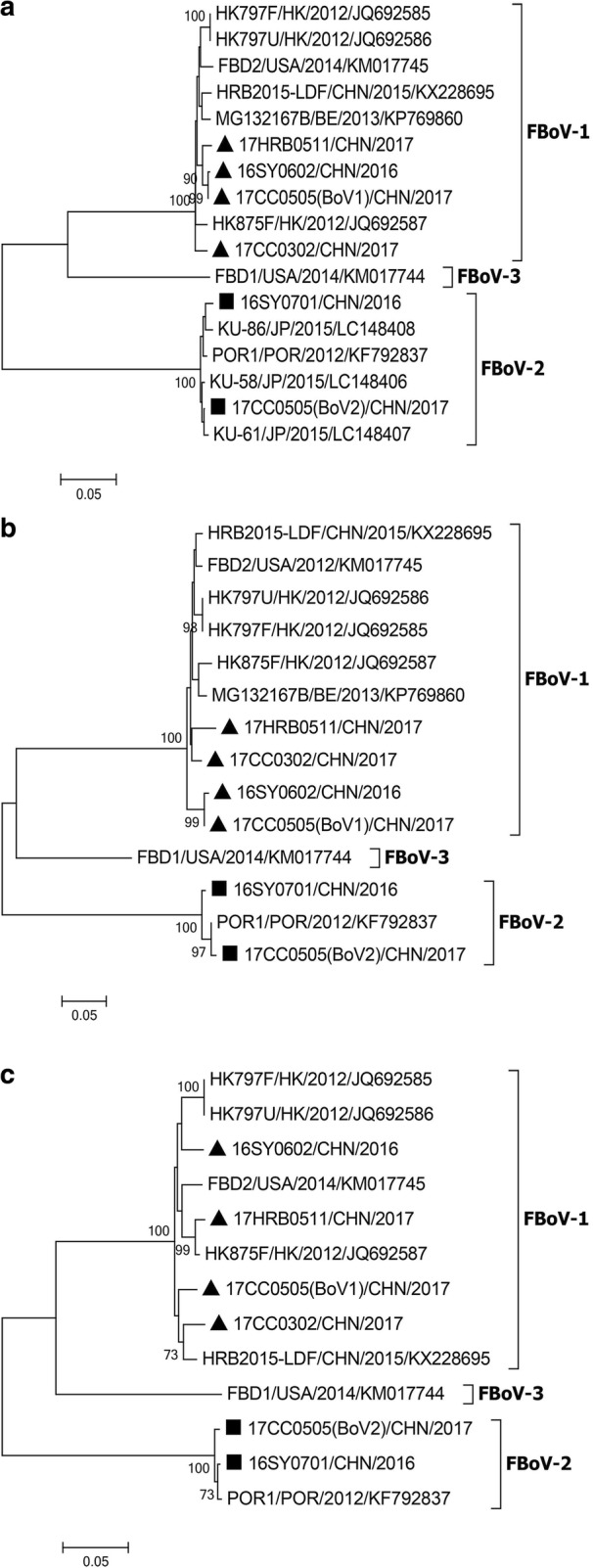
Phylogenetic tree of feline bocavirus based on the deduced amino acid sequences of genes for (**a**) NS1, (**b**) NP1 and (**c**) VP1. The tree is generated using the neighbor-joining method with 1000 bootstrap replicates, and only bootstrap values > 70% are displayed above the tree branches. The black triangles and black squares indicate FBoV-1 strains and FBoV-2 strains, respectively, identified in this study. FBoV, feline bocavirus. BE, Belgium; CHN, China; HK, HongKong; POR, Portugal; JP, Japan; USA, United States of America

The NJ phylogenetic tree and ML phylogenetic tree based on the full-length genome sequences of the 6 strains identified in this study and other bocavirus sequences from various animals were performed. These six strains were more closely related to other FBoV strains than to other bocaviruses, and belonged to two different genotypes (Fig. 4 and Additional file 5). Then, we analyzed the phylogenetic relationship of different FBoV strains based on the deduced amino acid sequences in the NS1, NP1 and VP1 regions. In the phylogenetic trees, all FBoV-2 strains, including 16SY0701 and 17CC0505(BoV2), cluster together in three different regions. The FBoV-1 strains divided into three lineages in the VP1 region (Fig. 5b), whereas these lineages were not clearly observed in the NS1 and NP1 regions (Fig. 5a/5c). Particularly, 16SY0602 and 17CC0505(BoV1) clustered together in the NS1 and NP1 trees, but were placed into different branches in the VP1 region (Fig. 5).

## Discussion

Feline bocavirus has previously been identified in fecal samples of cats with diarrhea in mainland China, but the prevalence and distribution of the genotypes are still unclear. In the current study, we first investigated the prevalence levels and genetic characteristics of FBoV in Northeast China, and analyzed the relationships between FBoV infection and gastrointestinal disease. The positive rate for FBoV was 25.9% in all fecal samples collected from Northeast China, which is higher than the positive rates previously reported in Hong Kong (7.2%, 26/263), Portugal (5.5%, 3/55), the USA (8.0%, 2/25) and Japan (9.9%, 10/101), even is almost 10-folds higher than the positive rates reported by Liu et al. in northeast China in 2015 [9, 18–20]. This may be mainly related to the source of samples. In our study, 63.9% (126/197) fecal samples were collected from cats in animal shelter center, the cross infection of FBoV in these cats might be the main reason for the high positive rate. Other factors, such as sample number, sampling time and the clinical status of the hosts, might also contribute to the difference in the positive rates. Moreover, 68.6% (35/51) of the FBoV-positive samples were identified as FBoV genotype 1, 23.5% (12/51) were classified as genotype 2, and 0.78% (4/51) were a mixed infection of FBoV-1 and FBoV-2, but no samples were identified as FBoV genotype 3. In previous reports, only FBoV-1 was detected in Hong Kong and mainland China [9, 21]. Our findings provide the first molecular evidence for the circulation of FBoV-1 and FBoV-2 among cats in China. Interestingly, FBoV-2 was not detected in Harbin, suggested that the geographical difference on the distribution of FBoV genotypes was existed in China. To further confirm this view, more investigations of the distribution of FBoV genotypes in China are needed in future study.

We analyzed the relationships between the FBoV infection and regions, season, clinical symptoms and sample source, and discovered that sample source and clinical symptoms were significantly related to the prevalence of FBoV. Our investigation indicated that the samples collected from cats with diarrhea displayed a higher positive rate for FBoV than those from normal cats. However, in a study by Takano, et al. [20], no significant association was found between FBoV infection and clinical symptoms, which differed from our findings. Most studies on the pathogenicity of other bocaviruses had confirmed that gastrointestinal and respiratory symptoms and severe systemic infection were caused by bocavirus in piglets, young dogs and human [8, 22, 23]. The higher prevalence of FBoV in cats with diarrhea in the present study suggests that FBoV is probably an enteric pathogen associated with diarrhea in cats, similar to other bocaviruses. Unfortunately, because of the limited sample number and sample information (including age, gender and breed) and the absence of screening for other enteric pathogens in FBoV-positive samples, the evidence that FBoV infection is associated with diarrhea is inadequate. Therefore, detailed investigations with an increased number of samples and scientific assessment of the role of coinfection with other enteric viruses would be helpful to further analyze the pathogenicity of FBoV. Furthermore, FBoVs were significantly more prevalent in cats from animal shelters (crowded environment) than that in cats from private veterinary clinics (relaxed environment), suggesting that a high-stress environment is a vital factor causing the circulation of FBoV.

NS1, as a major nonstructural protein, is vital for replication of bocavirus and is widely used for identification of the species and genotype of bocaviruses. Moreover, NS1 is also the most variable nonstructural protein in bocaviruses. We selected partial NS1 gene that could identify FBoV genotype and determine genetic diversity to construct phylogenetic tree. The phylogenetic analysis showed that the 24 sequences obtained in this study were divided into two clusters. Eight sequences identified as FBoV-2 by PCR clustered together and shared > 98% identities with FBoV2 reference strains from Portugal and Japan at the nucleotide and amino acid levels. However, the sixteen other sequences showed 96.3–98.6% nucleotide identities to the FBoV-1 references strains and formed two different groups. These analytical results revealed that genetic diversity is exhibited in FBoV strains in Northeast China. In the phylogenetic tree (Fig. 2), all Chinese FBoV-1 strains, except for 16CC0803, 16CC1105, 17CC0302 and 17CC0508, clustered together and exhibited 14 identical nucleotide replacements in the partial NS1 gene compared with the other FBoV-1 strains from different countries, suggesting that a novel FBoV-1 subgroup with a unique genetic evolution is circulating in Northeast China.

Six nearly complete FBoV genomes obtained in this study encoded three ORFs: NS1, NP1 and VP1/VP2, similar to other bocaviruses. The genomic organization was identical to that previously described by Lau et al. and Ng et al. [9, 19]. According to the existing criteria for bocavirus classification by ICTV, the strains belong to different genotypes if the amino acid identity of their full-length NS1 gene is < 95% [2]. In our study, 16SY0701 and 17CC0505(BoV2) have a > 98.8% identity compared with the FBoV-2 reference strain and < 70% identities compared with other genotype strains in the NS1 gene at the amino acid level, further demonstrating that these two strains belong to the FBoV genotype 2. The other four identified strains shared 96.9–97.8% amino acid identities with the FBoV-1 reference strains and were identified as the FBoV genotype 1. In the phylogenetic trees of the full-length genome for bocavirus, the six identified FBoV strains are closely related to other feline bocaviruses, and divided into different clusters according to their genotypes (Fig. 4). Due to the limited complete genome sequences of FBoV (5 for FBoV-1, 1 for FBoV-2 and 1 for FBoV-3) in GenBank, the genetic diversity in different geographical regions was not analyzed in the present study. We next analyzed the genetic similarity of the complete genome and the phylogenetic relationships based on the deduced amino acid sequences of NS1, NP1 and VP1 between identified FBoV strains and reference strains. For the two identified FBoV-2 strains, we found no significant difference of the complete genome and three ORFs at the nucleotide and amino acid levels compared with the reference strain POR1. However, lower similarities were exhibited in the NP1 and VP1 regions of the FBoV-1 strains identified in this study (Fig. 3). NP1 is a unique nonstructural protein for bocavirus, and is critical for viral replication [4, 24]. In the genome of FBoV, the NP1 gene shares a long overlap with the NS1 gene; therefore, the variation of NP1 may generate novel FBoV genotypes. The VP1 protein is a major structural protein in FBoV and plays a critical role in viral invasion and pathogenicity [25]. The genetic variation may influence the three-dimensional structure of the VP1 protein, which leads to the change of tissue tropism, antigenicity and pathogenicity for FBoV [26]. In our study, the effect of these mutations in the NP1 and VP1 regions of the FBoV-1 strains needs to be further analyzed via massive experimental and scientific predictions of protein structure and function. Furthermore, these data further demonstrate that the NS1 gene is more highly conserved than the NP1 and VP1 genes of FBoV.

Recombination plays a critical role in viral evolutionary processes to generate new genotypes. Recombination among different strains in the same genotype or different genotypess strains of bocavirus have been reported in previous papers [26–29]. Yang et al., reported a novel PBoV strain, PBoV3C, with a recombination breakpoint at the boundary between conserved and non-conserved regions in the NP1 gene [26]. In an investigation of CBoV by Guo et al., the recombination events among different subgroups of CBoV-2 occurred frequently in the VP2 gene of CBoV in China [28]. A large number of investigations showed that the coinfection of different genotypes viruses in the same host increased the probability of genetic reombination. In our study, four samples were identified with coinfection of FBoV-1 and FBoV-2. We analyzed the recombination events among different genotype strains, 17CC0505(BoV1) and 17CC0505(BoV2) isolated from the same sample and 4 other identified strains and reference strains, but no recombination breakpoint was found in all FBoV strains. To better monitor recombination events in FBoV, an extensive molecular epidemiological investigation in more regions will be helpful for further study.

This study is subject to several limitations. First, we investigated the prevalence and genetic characteristics of FBoVs identified from fecal samples in the present study, but the investigation of the prevalence of FBoV in other samples, such as nasal swabs, blood and tissue samples, were not performed, because of the limitation of sample collection. Second, we failed to isolate and culture FBoVs from the fresh feces of FBoV-positive samples. Thus, we were not able to explicitly assess the pathogenicity of FBoV. Furthermore, we only detected FBoV in all samples, but other enteric pathogens in FBoV-positive samples were not tested. This also limit the assessment of the relationship between FBoV infection and diarrhea in cats. In future studies, we will perform the detailed investigations of the prevalence of FBoV in different samples that collected from more regions in China to further analyze the prevalence and pathogenicity of FBoV.

## Conclusions

In conclusion, this study demonstrates that FBoV-1 and FBoV-2 widely circulate in cats in Northeast China and that FBoV-1 is more prevalent. The high prevalence of FBoV in cats with diarrhea symptoms suggests that FBoV infection may be associated with diarrhea in cats. The FBoV strains identified from Northeast China exhibit considerable genetic diversity based on the complete genome and encoding gene. Furthermore, the complete genomes of 4 FBoV-1 strains and 2 FBoV-2 strains were successfully sequenced from FBoV-positive samples, and detailed genetic characterization was described in this study. Our findings not only provide more gene sequences for investigation of the molecular epidemiology of FBoV worldwide, but also contribute to further study of the prevalence, genotype distribution and genetic diversity of FBoV in China.

## Additional files

Additional file 1: List of representative bocavirus strains obtained from the GenBank database for genetic analysis and phylogenetic tree construction. (DOC 64 kb)

Additional file 2: Summary of detailed clinical informations for the 51 FBoV-positive samples identified in the present study. M, male; FM, female; PVC, private veterinary clinic; ASC, animal shelter center. (DOC 98 kb)

Additional file 3: A maximum-likelihood phylogenetic tree based on partial NS1 gene (705 nt) of feline bocavirus. The black triangles indicate strains belonging to FBoV-1 identified in this study, and the black squares indicate strains belonging to FBoV-2 identified in the present study. (TIF 635 kb)

Additional file 4: Comparison of nucleotide and amino acid sequence identity in the complete genome and three ORFs of different feline bocavirus genotypes. Nucleotide similarity (%)/ deduced amino acid similarity (%) is shown in the table. Six strains identified in this study were compared to each other and were compared with other reference strains of different FBoV genotypes based on the complete genome and three ORFs. Bold text indicates that the similarity between identified strains and reference strains is higher than 85%. (DOC 62 kb)

Additional file 5: Phylogenetic tree based on the complete genome of bocaviruses. The phylogenetic analysis was performed using maximum-likelihood method in MEGA 7.0 software. The black triangles and black squares indicate FBoV-1 strains and FBoV-2 strains, respectively, identified in this study. (TIF 1023 kb)

## Abbreviations

aa: amino acid
BPV: Bovine parvovirus
CBoV: Canine bocavirus
CslBoV: California sea lion bocavirus
FBoV: Feline bocavirus
GBoV: Gorilla bocavirus
HBoV: Human bocavirus
MVC: Canine minute virus
nt: Nucleotide
ORF: Open reading frame
PBoV: Porcine bocavirus
PCR: Polymerase chain reaction
