# Supplementary material for: Detection and genetic characterization of feline bocavirus in Northeast China
Source: Virol J. 2018 Aug 8;15:125. doi: 10.1186/s12985-018-1034-3 (PMC6083571; doi:10.1186/s12985-018-1034-3)
Supplement: Supplementary file 1 — List of representative bocavirus strains obtained from the GenBank database for genetic analysis and phylogenetic tree construction. (DOC 64 kb) [file 12985_2018_1034_MOESM1_ESM.doc]

**Additional file 1** List of representative bocavirus strains obtained from the GenBank database for genetic analysis and phylogenetic tree construction.

| **No.** | **Strain** | **Host** | **Genotype** | **Country** | **Year** | **Accession no.** |
| --- | --- | --- | --- | --- | --- | --- |
| 1 | HK797F | Cat | FBoV-1 | HongKong | 2012 | JQ692585 |
| 2 | HK797U | Cat | FBoV-1 | HongKong | 2012 | JQ692586 |
| 3 | HK875F | Cat | FBoV-1 | HongKong | 2012 | JQ692587 |
| 4 | MG132167B | Cat | FBoV-1 | Belgium | 2013 | KP769860 |
| 5 | FBD2 | Cat | FBoV-1 | USA | 2014 | KM017745 |
| 6 | HRB2015-LDF | Cat | FBoV-1 | China | 2015 | KX228695 |
| 7 | POR1 | Cat | FBoV-2 | Portugal | 2012 | KF792837 |
| 8 | KU-89 | Cat | FBoV-2 | Japan | 2015 | LC148408 |
| 9 | KU-58 | Cat | FBoV-2 | Japan | 2015 | LC148406 |
| 10 | KU-61 | Cat | FBoV-2 | Japan | 2015 | LC148407 |
| 11 | FBD1 | Cat | FBoV-3 | USA | 2014 | KM017744 |
| 12 | mvc | Dog | MVC | USA | 2002 | AF495467 |
| 13 | SH1 | Dog | MVC | China | 2010 | FJ899734 |
| 14 | CON-161 | Dog | CBoV-1 | USA | 2010 | JN648103 |
| 15 | UCD | Dog | CBoV-3 | USA | 2011 | KC580640 |
| 16 | HK891F | Dog | CBoV | HongKong | 2012 | JQ692591 |
| 17 | 13D003 | Dog | CBoV | South Korea | 2013 | KP281713 |
| 18 | 14Q216 | Dog | CBoV | South Korea | 2014 | KP281720 |
| 19 | GZHD15 | Dog | CBoV-2 | China | 2016 | KY038922 |
| 20 | 1153 | California sea lion | CslBoV-1 | USA | 2010 | JN420361 |
| 21 | 9822 | California sea lion | CslBoV-2 | USA | 2010 | JN420366 |
| 22 | YNJH | Bat | BatBoV | China | 2016 | KT592508 |
| 23 | 64-1 | Pig | PBoV-3 | UK | 2004 | JF512472 |
| 24 | ZJD-1 | Pig | PBoV-1 | China | 2006 | HM053693 |
| 25 | ZJD-2 | Pig | PBoV-2 | China | 2006 | HM053694 |
| 26 | SH17N-1 | Pig | PBoV-4 | HongKong | 2011 | JF429835 |
| 27 | HK1S | Rat | RatBoV | HongKong | 2010 | KT454512 |
| 28 | HK2TV | Rat | RatBoV | HongKong | 2010 | KT454516 |
| 29 | bpv | Cattle | BPV | USA | 1987 | M14363 |
| 30 | st2 | Human | HBoV-1 | USA | 2005 | DQ000496 |
| 31 | W471 | Human | HBoV-3 | Australia | 2009 | EU918736 |
| 32 | PK-5510 | Human | HBoV-2 | Pakistan | 2014 | FJ170728 |
| 33 | NI-385 | Human | HBoV-4 | USA | 2014 | FJ973561 |
