# Supplementary material for: Detection and genetic characterization of feline bocavirus in Northeast China
Source: Virol J. 2018 Aug 8;15:125. doi: 10.1186/s12985-018-1034-3 (PMC6083571; doi:10.1186/s12985-018-1034-3)
Supplement: Supplementary file 2 — Summary of detailed clinical informations for the 51 FBoV-positive samples identified in the present study. M, male; FM, female; PVC, private veterinary clinic; ASC, animal shelter center. (DOC 98 kb) [file 12985_2018_1034_MOESM2_ESM.doc]

**Additional file 2** Summary of detailed clinical informations for the 51 FBoV-positive samples identified in the present study.

| ID | Age | Gender | Year | Region | Source | Clinical symptom | Genotype of FBoV | sequencing | Accession no. |
| --- | --- | --- | --- | --- | --- | --- | --- | --- | --- |
| 16SY0602 | - | - | 2016 | Shenyang | ASC | Diarrhea | FBoV-1 | Partial NS1 gene  Complete genome | MH155946 |
| 16SY0701 | 1 m | M | 2016 | Shenyang | PVC | Normal | FBoV-2 | Partial NS1 gene  Complete genome | MH155950 |
| 16SY0707 | - | - | 2016 | Shenyang | ASC | Diarrhea | FBoV-1 | - |  |
| 16SY0714 | - | - | 2016 | Shenyang | ASC | Diarrhea | FBoV-1 | - |  |
| 16SY0715 | 2 m | M | 2016 | Shenyang | PVC | Diarrhea | FBoV-1 | Partial NS1 gene | MH155935 |
| 16SY0720 | 2 m | FM | 2016 | Shenyang | PVC | Diarrhea | FBoV-1 | - |  |
| 16JZ0602 | 2 m | FM | 2016 | Jinzhou | PVC | Diarrhea | FBoV-1 | - |  |
| 16JZ0607 | 2 m | FM | 2016 | Jinzhou | PVC | Diarrhea | FBoV-1 | Partial NS1 gene | MH155932 |
| 16JZ0613 | - | - | 2016 | Jinzhou | ASC | Diarrhea | FBoV-2 | Partial NS1 gene | MH155933 |
| 16JZ0702 | - | - | 2016 | Jinzhou | ASC | Normal | FBoV-1 | Partial NS1 gene | MH155934 |
| 16CC0801 | - | - | 2016 | Changchun | ASC | Diarrhea | FBoV-2 | - |  |
| 16CC0803 | 1 m | M | 2016 | Changchun | PVC | Normal | FBoV-1 | Partial NS1 gene | MH155928 |
| 16CC0805 | 2 m | M | 2016 | Changchun | PVC | Diarrhea | FBoV-1 | - |  |
| 16CC1101 | - | - | 2016 | Changchun | ASC | Diarrhea | FBoV-1 | - |  |
| 16CC1103 | - | - | 2016 | Changchun | ASC | Diarrhea | FBoV-2 | Partial NS1 gene | MH155929 |
| 16CC1105 | - | - | 2016 | Changchun | ASC | Normal | FBoV-1 | Partial NS1 gene | MH155930 |
| 16CC1106 | - | - | 2016 | Changchun | ASC | Diarrhea | FBoV-1 | - |  |
| 16CC1107 | - | - | 2016 | Changchun | ASC | Normal | FBoV-2 | - |  |
| 16CC1109 | - | - | 2016 | Changchun | ASC | Diarrhea | FBoV-1 | - |  |
| 16JL0804 | - | - | 2016 | Jilin | ASC | Normal | FBoV-1 | Partial NS1 gene | MH155931 |
| 17SY0602 | - | - | 2017 | Shenyang | ASC | Normal | FBoV-2 | - |  |
| 17SY1201 | - | - | 2017 | Shenyang | ASC | Diarrhea | FBoV-1 | - |  |
| 17SY1205 | - | - | 2017 | Shenyang | ASC | Normal | FBoV-1 | - |  |
| 17CC0302 | 3 m | FM | 2017 | Changchun | ASC | Diarrhea | FBoV-1 | Partial NS1 gene  Complete genome | MH155947 |
| 17CC0308 | 3 m | FM | 2017 | Changchun | ASC | Diarrhea | FBoV-1 | - |  |
| 17CC0311 | 3 m | M | 2017 | Changchun | ASC | Diarrhea | FBoV-1 | - |  |
| 17CC0505 | 2 m | M | 2017 | Changchun | ASC | Diarrhea | FBoV-1+FBoV-2 | Partial NS1 gene for FBoV-1and -2  Complete genome for FBoV-1and -2 | MH155949  MH155951 |
| 17CC0507 | 1 m | FM | 2017 | Changchun | PVC | Normal | FBoV-2 | Partial NS1 gene | MH155936 |
| 17CC0508 | - | - | 2017 | Changchun | ASC | Diarrhea | FBoV-1 | Partial NS1 gene | MH155937 |
| 17CC0704 | 1 m | M | 2017 | Changchun | ASC | Normal | FBoV-1+FBoV-2 | Partial NS1 gene for FBoV-1and -2 | MH155938 |
| 17CC0708 | 1 m | FM | 2017 | Changchun | ASC | Diarrhea | FBoV-1 | - | MH155939 |
| 17CC0709 | 1 m | M | 2017 | Changchun | ASC | Diarrhea | FBoV-1 | - |  |
| 17CC0805 | - | - | 2017 | Changchun | ASC | Normal | FBoV-2 | - |  |
| 17CC0809 | 1 m | FM | 2017 | Changchun | PVC | Normal | FBoV-1 | Partial NS1 gene | MH155940 |
| 17CC0810 | - | - | 2017 | Changchun | ASC | Normal | FBoV-2 | - |  |
| 17CC1105 | 1 m | M | 2017 | Changchun | ASC | Diarrhea | FBoV-1 | - |  |
| 17CC1108 | 2 m | FM | 2017 | Changchun | ASC | Diarrhea | FBoV-1+FBoV-2 | Partial NS1 gene for FBoV-1and -2 | MH155941 |
| 17CC1112 | 2 m | FM | 2017 | Changchun | ASC | Diarrhea | FBoV-1+FBoV-2 | - | MH155942 |
| 17CC1113 | 3m | FM | 2017 | Changchun | ASC | Diarrhea | FBoV-1 | - |  |
| 17JL0301 | 1 m | M | 2017 | Jilin | PVC | Normal | FBoV-1 | - |  |
| 17JL0311 | - | - | 2017 | Jilin | ASC | Diarrhea | FBoV-2 | - |  |
| 17JL0312 | - | - | 2017 | Jilin | ASC | Diarrhea | FBoV-2 | Partial NS1 gene | MH155944 |
| 17JL0315 |  | - | 2017 | Jilin | ASC | Diarrhea | FBoV-2 | - |  |
| 17JL0317 | - | - | 2017 | Jilin | ASC | Diarrhea | FBoV-1 | Partial NS1 gene | MH155945 |
| 17JL0318 | - | - | 2017 | Jilin | ASC | Diarrhea | FBoV-1 | - |  |
| 17JL0506 | 2 m | M | 2017 | Jilin | ASC | Diarrhea | FBoV-1 | - |  |
| 17HRB0503 | 6 m | M | 2017 | Harbin | PVC | Diarrhea | FBoV-1 | - |  |
| 17HRB0511 | 2 m | FM | 2017 | Harbin | PVC | Diarrhea | FBoV-1 | Partial NS1 gene  Complete genome | MH155948 |
| 17HRB0903 | 3 m | - | 2017 | Harbin | ASC | Diarrhea | FBoV-1 | - |  |
| 17HRB0909 | 6 m | - | 2017 | Harbin | ASC | Normal | FBoV-1 | - |  |
| 17HRB0910 | 6 m | - | 2017 | Harbin | ASC | Normal | FBoV-1 | Partial NS1 gene | MH155943 |

M, male; FM, female; PVC, private veterinary clinic; ASC, animal shelter center.
