# Supplementary figures and images for: Detection and genetic characterization of feline bocavirus in Northeast China
Source: Virol J. 2018 Aug 8;15:125. doi: 10.1186/s12985-018-1034-3 (PMC6083571; doi:10.1186/s12985-018-1034-3)

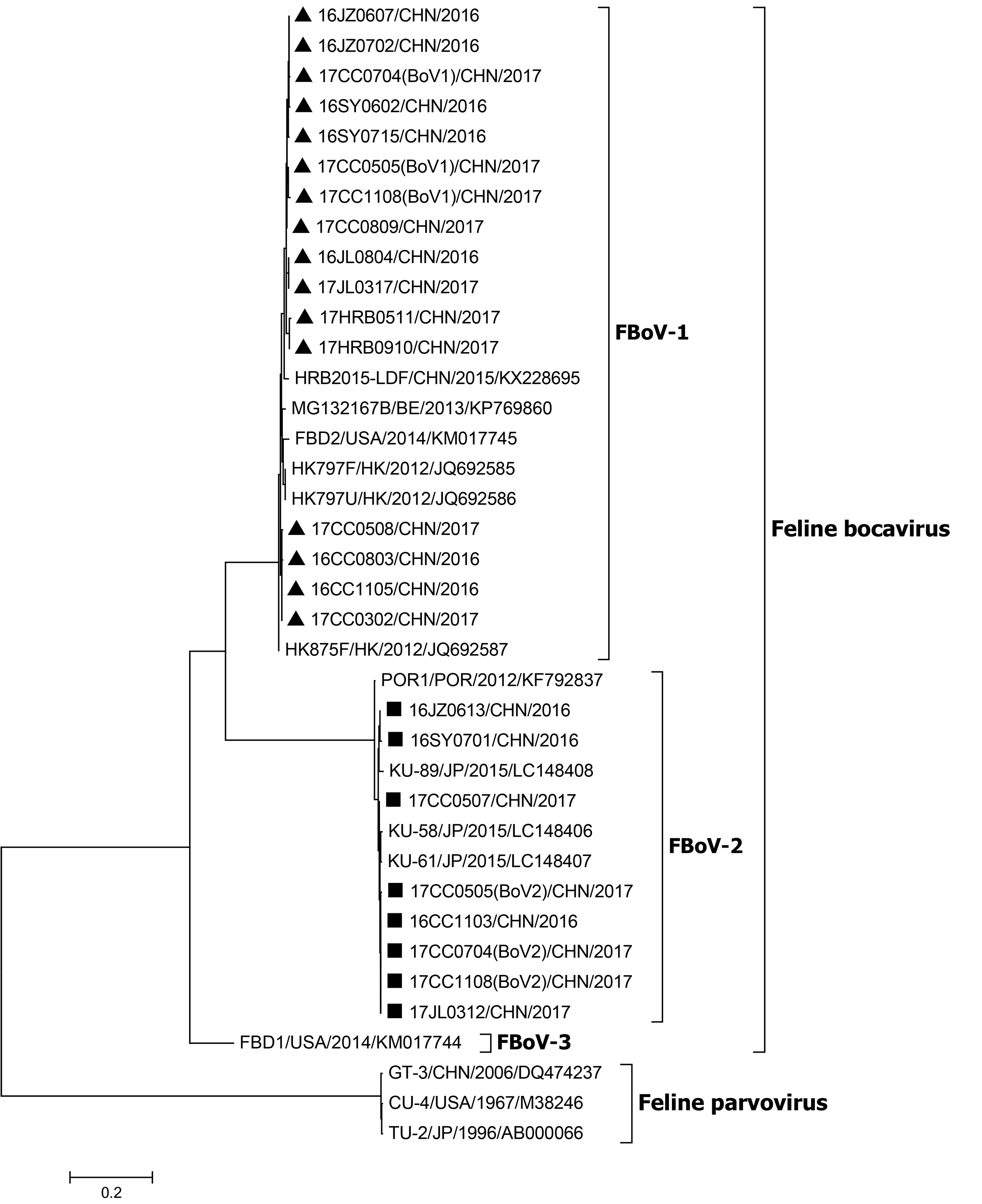

Supplement: Supplementary file 3 — A maximum-likelihood phylogenetic tree based on partial NS1 gene (705 nt) of feline bocavirus. The black triangles indicate strains belonging to FBoV-1 identified in this study, and the black squares indicate strains belonging to FBoV-2 identified in the present study. (TIF 635 kb) [file 12985_2018_1034_MOESM3_ESM.tif]

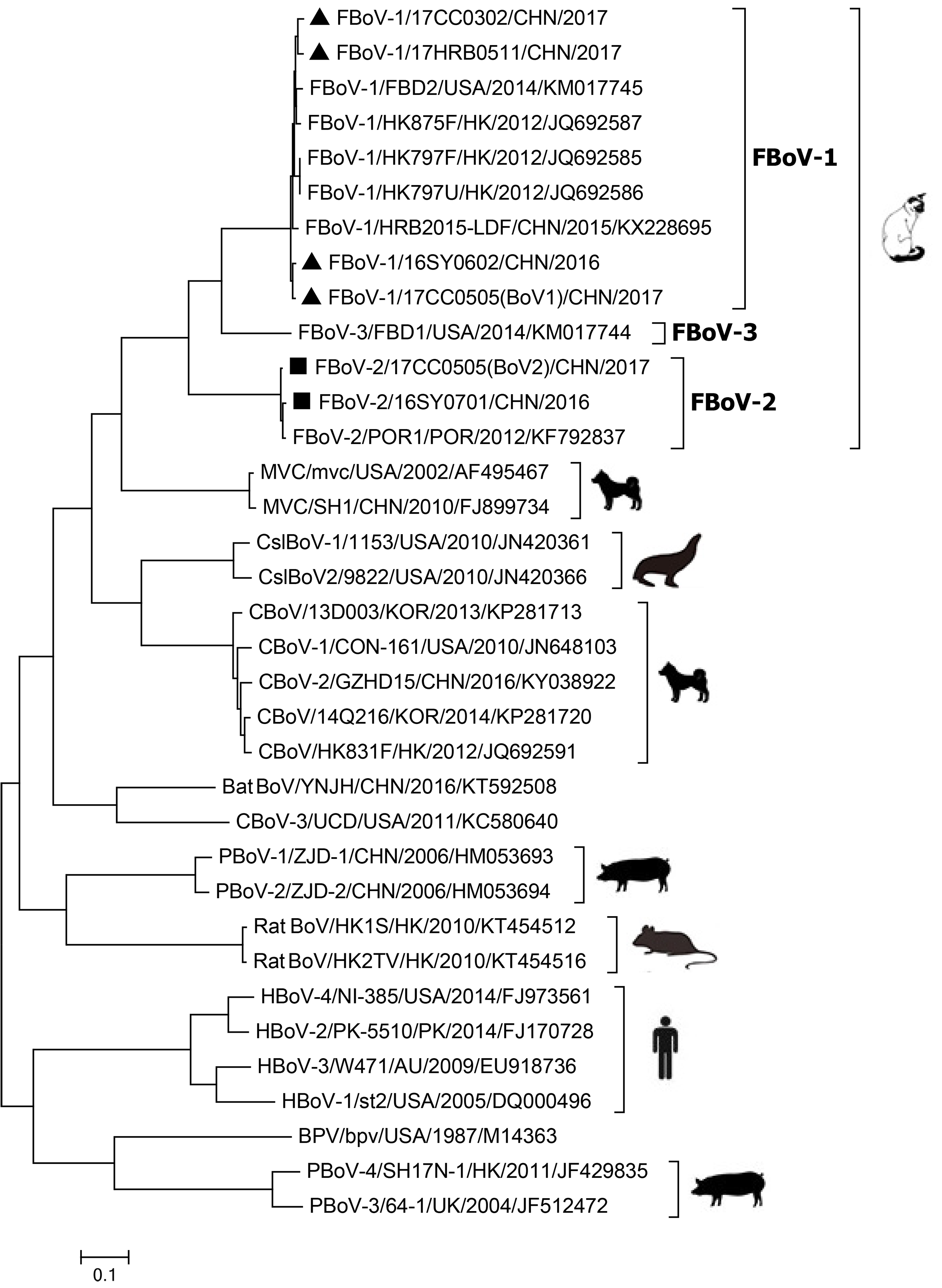

Supplement: Supplementary file 5 — Phylogenetic tree based on the complete genome of bocaviruses. The phylogenetic analysis was performed using maximum-likelihood method in MEGA 7.0 software. The black triangles and black squares indicate FBoV-1 strains and FBoV-2 strains, respectively, identified in this study. (TIF 1023 kb) [file 12985_2018_1034_MOESM5_ESM.tif]
