# Supplementary material for: Detection and genetic characterization of feline bocavirus in Northeast China
Source: Virol J. 2018 Aug 8;15:125. doi: 10.1186/s12985-018-1034-3 (PMC6083571; doi:10.1186/s12985-018-1034-3)
Supplement: Supplementary file 4 — Comparison of nucleotide and amino acid sequence identity in the complete genome and three ORFs of different feline bocavirus genotypes. Nucleotide similarity (%)/ deduced amino acid similarity (%) is shown in the table. Six strains identified in this study were compared to each other and were compared with other reference strains of different FBoV genotypes based on the complete genome and three ORFs. Bold text indicates that the similarity between identified strains and reference strains is higher than 85%. (DOC 62 kb) [file 12985_2018_1034_MOESM4_ESM.doc]

**Additional file 4** Comparison of nucleotide and amino acid sequence identity in the complete genome and three ORFs of different feline bocavirus genotypes.

|  | Strains idetified in this study | | | | | |  | Reference strains | | | |
| --- | --- | --- | --- | --- | --- | --- | --- | --- | --- | --- | --- |
|  | FBoV-1 | | | | FBoV-2 | |  | FBoV-1 | | FBoV-2 | FBoV-3 |
|  | 16SY0602 | 17HRB0511 | 17CC0302 | 17CC0505  (BoV1) | 16SY0701 | 17CC0505  (BoV2) |  | HK797U  JQ692586 | HRB2015-LDF  KX228698 | POR1  KF792837 | FBD1  KM017744 |
| 16SY0602 |  |  |  |  |  |  |  |  |  |  |  |
| Complete genome | 100/100 | 97.3/95.2 | 96.8/95.0 | 98.7/97.0 | 67.0/53.0 | 67.4/53.7 |  | **97.3/95.3** | **97.3/94.9** | 67.2/53.5 | 74.2/62.2 |
| ORF1 (NS1) | 100/100 | 98.1/98.3 | 97.2/97.8 | 99.7/99.9 | 61.3/67.3 | 61.4/61.8 |  | **97.1/97.1** | **97.9/97.8** | 61.4/67.8 | 66.6/76.5 |
| ORF3 (NP1) | 100/100 | 97.0/95.0 | 97.0/95.4 | 99.8/99.5 | 70.5/63.1 | 70.7/62.2 |  | **96.7/95.9** | **97.3/95.9** | 70.7/62.7 | 77.3/71.2 |
| ORF2 (VP1/VP2) | 100/100 | 96.3/95.2 | 95.9/95.1 | 97.3/96.9 | 70.7/70.7 | 70.9/70.8 |  | **96.9/96.8** | **95.5/94.9** | 70.9/70.7 | 75.7/78.1 |
| 17HRB0511 |  |  |  |  |  |  |  |  |  |  |  |
| Complete genome | 97.3/95.2 | 100/100 | 97.4/95.5 | 97.3/94.9 | 67.0/52.9 | 67.0/53.3 |  | **96.9/94.9** | **97.2/94.6** | 67.0/53.0 | 73.7/61.4 |
| ORF1 (NS1) | 98.1/98.3 | 100/100 | 97.8/97.1 | 98.1/98.4 | 61.2/67.3 | 61.1/62.1 |  | **96.9/96.9** | **97.7/97.4** | 61.4/67.8 | 66.6/76.9 |
| ORF3 (NP1) | 97.0/95.0 | 100/100 | 96.5/95.9 | 97.1/95.4 | 70.5/62.7 | 70.7/61.8 |  | **96.7/95.9** | **97.3/96.3** | 70.7/62.2 | 77.3/69.9 |
| ORF2 (VP1/VP2) | 96.3/95.2 | 100/100 | 96.9/95.7 | 96.1/95.4 | 70.7/71.0 | 70.6/71.1 |  | **96.6/96.4** | **95.8/95.6** | 70.4/70.5 | 74.8/77.5 |
| 17CC0302 |  |  |  |  |  |  |  |  |  |  |  |
| Complete genome | 96.8/95.0 | 97.4/95.5 | 100/100 | 97.1/95.2 | 67.1/53.3 | 67.3/53.8 |  | **97.0/95.4** | **97.4/95.7** | 67.2/53.6 | 73.9/61.6 |
| ORF1 (NS1) | 97.2/97.8 | 97.8/97.1 | 100/100 | 97.4/97.9 | 61.6/67.5 | 61.3/61.8 |  | **97.3/97.5** | **97.3/97.3** | 61.6/68.1 | 66.9/76.6 |
| ORF3 (NP1) | 97.0/95.4 | 96.5/95.9 | 100/100 | 97.1/95.9 | 70.8/63.6 | 71.162.7 |  | **97.9/97.3** | **97.4/96.8** | 71.0/63.1 | 77.8/72.1 |
| ORF2 (VP1/VP2) | 95.9/95.1 | 96.9/95.7 | 100/100 | 96.3/95.9 | 70.8/70.7 | 70.9/70.8 |  | **96.2/95.1** | **96.7/96.9** | 70.9/71.1 | 75.0/78.8 |
| 17CC0505(BoV1) |  |  |  |  |  |  |  |  |  |  |  |
| Complete genome | 98.7/97.0 | 97.3/94.9 | 97.1/95.2 | 100/100 | 67.0/53.2 | 67.4/53.8 |  | **97.1/94.6** | **97.8/95.9** | 67.1/53.3 | 74.1/61.8 |
| ORF1 (NS1) | 99.7/99.9 | 98.1/98.4 | 97.4/97.9 | 100/100 | 61.3/67.3 | 61.3/61.8 |  | **97.4/97.5** | **98.1/97.3** | 61.3/68.1 | 66.5/76.6 |
| ORF3 (NP1) | 99.8/99.5 | 97.1/95.4 | 97.1/95.9 | 100/100 | 70.7/63.6 | 71.0/62.7 |  | **97.9/96.3** | **97.9/96.3** | 70.8/63.1 | 77.3/71.2 |
| ORF2 (VP1/VP2) | 97.3/96.9 | 96.1/95.4 | 96.3/95.9 | 100/100 | 70.7/71.1 | 71.1/71.2 |  | **96.2/95.7** | **96.8/96.9** | 70.8/70.7 | 75.4/77.5 |
| 16SY0701 |  |  |  |  |  |  |  |  |  |  |  |
| Complete genome | 67.0/53.0 | 97.3/94.9 | 67.1/53.3 | 67.0/53.2 | 100/100 | 98.9/98.6 |  | 67.2/53.2 | 67.3/53.2 | **99.0/98.9** | 66.8/53.0 |
| ORF1 (NS1) | 61.3/67.3 | 98.1/98.4 | 61.6/67.5 | 61.3/67.3 | 100/100 | 98.5/98.9 |  | 61.5/67.4 | 61.8/67.0 | **98.3/98.9** | 64.7/67.8 |
| ORF3 (NP1) | 70.5/63.1 | 97.1/95.4 | 70.8/63.6 | 70.7/63.6 | 100/100 | 98.1/98.3 |  | 71.3/63.6 | 71.1/63.6 | **98.8/98.7** | 71.3/67.0 |
| ORF2 (VP1/VP2) | 70.7/70.7 | 96.1/95.4 | 70.8/70.7 | 70.7/71.1 | 100/100 | 99.4/99.0 |  | 70.9/71.2 | 69.8/70.1 | **99.6/99.6** | 69.6/69.8 |
| 17CC0505(BoV2) |  |  |  |  |  |  |  |  |  |  |  |
| Complete genome | 67.4/53.7 | 67.0/53.3 | 67.3/53.8 | 67.4/53.8 | 98.9/98.6 | 100/100 |  | 67.4/53.8 | 67.5/53.9 | **98.9/98.8** | 66.9/53.2 |
| ORF1 (NS1) | 61.4/61.8 | 61.1/62.1 | 61.3/61.8 | 61.3/61.8 | 98.5/98.9 | 100/100 |  | 61.2/61.7 | 61.5/61.8 | **98.2/98.8** | 60.6/61.7 |
| ORF3 (NP1) | 70.7/62.2 | 70.7/61.8 | 71.162.7 | 71.0/62.7 | 98.1/98.3 | 100/100 |  | 71.7/62.7 | 71.4/62.7 | **99.3/99.6** | 71.0/67.9 |
| ORF2 (VP1/VP2) | 70.9/70.8 | 70.6/71.1 | 70.9/70.8 | 71.1/71.2 | 99.4/99.0 | 100/100 |  | 71.0/71.1 | 70.1/69.9 | **99.4/99.2** | 69.8/70.0 |

Nucleotide similarity (%)/ deduced amino acid similarity (%) is shown in the table. Six strains identified in this study were compared to each other and were compared with other reference strains of different FBoV genotypes based on the complete genome and three ORFs. Bold text indicates that the similarity between identified strains and reference strains is higher than 85% .
